# Circulating Inhibitor against Factor X: A Rare Cause of Hemorrhagic Diathesis

**DOI:** 10.1155/2023/5510654

**Published:** 2023-12-13

**Authors:** P. Rossignon, F. Grandjean, A. Claessens, N. Weynants

**Affiliations:** ^1^Hematology-Oncology Department and Cardiology Department, Centre Hospitalier de Luxembourg, Luxembourg City, Luxembourg; ^2^Department of Internal Medicine, Erasmus University Hospital, ULB, Brussels, Belgium; ^3^Department of Clinical Biology, Department of Endocrinology and Diabetology and Department of Hematology and Oncology, Cliniques du Sud Luxembourg-Vivalia, Arlon, Belgium

## Abstract

Acquired coagulopathies resulting from factor X deficiency are rare and typically associated with amyloidosis or plasma cell dyscrasia. Factor X plays a pivotal role in the coagulation cascade, converting prothrombin into thrombin and facilitating the formation of fibrinogen and thrombus. While its occurrence following common infections is extremely rare, isolated cases have been documented. We present a rare case of bleeding diathesis in a patient with community-acquired pneumonia, where prolonged activated partial thromboplastin time (aPTT) and prothrombin time (PT) led to the diagnosis of an infectious-triggered acquired circulating inhibitor targeting factor X. Prompt treatment with methylprednisolone effectively controlled the inhibitor without recurrence. This case report provides insights into the diagnostic strategies, differential algorithm, and therapeutic approaches for managing this rare coagulopathy.

## 1. Introduction

Secondary hemostasis aims to form a fibrin-platelet thrombus through the sequential activation of various clotting factors. Deficiencies in these factors can result in a wide range of clinical and biological implications, which vary based on the specific role and position of the factors within the coagulation cascade.

Factor X plays a pivotal role in the coagulation cascade by activating prothrombin to thrombin, which is a critical step in the conversion of fibrinogen and the formation of thrombus. While acquired deficiencies in factor X often involve multiple factors, such as in cases of liver diseases or vitamin K deficiencies [1], isolated factor X deficiency is typically congenital, known as Stuart–Prower disease, with an incidence of approximately 1 in 1,500,000 [2]. However, acquired isolated factor X deficiency (AiF10D) is an extremely rare condition, with only a few reported cases in the literature. Notably, among these reported cases, the majority are associated with AL amyloidosis. Instances of AiF10D without amyloidosis are exceptionally rare, with only a few documented cases. A minority of these cases (e.g., twenty-one cases) have demonstrated the presence of a circulating inhibitor specifically targeting factor X, strongly suggesting an association with factor X-specific autoantibodies. This report presents a rare case of acquired coagulopathy due to factor X deficiency, with evidence of a specific inhibitor directed against this protein, occurring in the context of a respiratory tract infection.

## 2. Case Description

A 33-year-old male with poorly controlled type 1 diabetes and a history of vitiligo and previous tonsillectomy was hospitalized with community-acquired lobar pneumonia. Initial laboratory investigations showed an inflammatory syndrome without significant leukocytosis. Coagulation tests revealed prolonged PT and aPTT, while fibrinogen level was normal (Table 1). The patient was promptly started on amoxicillin-clavulanic acid for pneumonia treatment. The following day, he developed visually striking signs of bleeding diathesis, including epistaxis, gingival bleeding, and gross hematuria. Despite these manifestations, he remained hemodynamically stable without significant hemoglobin loss or deficits in peripheral perfusion.

Detailed history taking did not reveal any significant bleeding events in the past. The tonsillectomy was uneventful, and there were no suggestive findings in the family history. The patient denied any substance abuse. Physical examination did not reveal any additional bleeding lesions.

### 2.1. Biological Investigations of Coagulation

The prolonged PT indicates a defect in the extrinsic pathway, while the prolonged aPTT indicates a defect in the intrinsic pathway. Simultaneous prolongation of both tests raises the possibility of combined pathway defects or a common pathway defect. This suggests the presence of abnormalities affecting multiple coagulation factors or a defect in a factor or mechanism that is essential for both pathways to function properly, i.e., that is part of the common pathway.

Potential causes for combined pathway defects include deficiencies of multiple factors, presence of circulating inhibitors (antibodies and antithrombotic drugs), or deficits associated with liver diseases, disseminated intravascular coagulation (DIC), or vitamin K deficiency [1] (Table 2).

For a common pathway defect, possibilities also include the presence of an inhibitor, or isolated deficiencies of factor V, factor X, prothrombin, or fibrinogen.

To investigate the underlying cause of the patient's bleeding diathesis, a plasma mixing study and coagulation factor assays were performed. Treatment with vitamin K and prothrombin complex concentrate (PCC) was initiated pending the results, but there was no clinical or laboratory improvement.

Simultaneously, further laboratory testing was conducted, including comprehensive autoimmune disorder panels, liver function tests, and assessments of immunoglobulin and complement levels, yielding normal results.

### 2.2. Identification of Circulating Inhibitor and Differential Diagnosis

The plasma mixing study failed to fully correct the coagulation times as expressed by a Rosner index of 15%, suggesting the presence of a circulating inhibitor (Table 3).

The Rosner index is an objective assessment method used during mixing tests. It is an indicator of circulating anticoagulants and is among the recommended techniques for interpreting mixing test results [3]. It is calculated using the formula (aPTT plasma mix − aPTT control)/aPTT patient, with values greater than 11% typically considered suggestive of an inhibitor effect [4].

Furthermore, coagulation factor assays revealed an isolated factor X decreased activity, initially measured at 18% and subsequently reconfirmed (Table 1).

At this point, the diagnostic possibilities included a specific antibody against factor X or lupus anticoagulant (LA).

LA is an autoantibody commonly associated with diverse autoimmune disorders, particularly systemic lupus erythematosus. It is referred to as an “anticoagulant” because it binds to phospholipids, crucial components for blood coagulation, thereby interfering with the normal clotting process and resulting in a prolonged aPTT.

Since coagulation factor assays inherently rely on the presence of phospholipids, the presence of LA can artificially underestimate the activity of factors. In this case, considering the significant prolongation of the activated aPTT, one would typically expect to observe decreased levels of several factors involved in the intrinsic pathway, if LA were present. However, this expected pattern was not observed.

In addition, it is important to note that the reference test used to detect LA, called diluted Russell's viper venom (DRVVT), is not validated if there are suspected circulating inhibitors against factor X [5, 6]. This is because DRVVT aims to activate factor X itself, which can be affected by such inhibitors. Furthermore, the diagnosis of LA requires the exclusion of other potential causes for prolonged aPTT, such as isolated defects in coagulation factors. Based on the clinical presentation and laboratory findings, LA was deemed less likely in this case. Negative results were obtained from the required second test for confirming LA, typically performed three months later, as well as tests for anticardiolipin antibodies and anti-*β*2 glycoprotein 1 antibodies (i.e., antiphospholipid antibodies). Since these situations did not meet the criteria for LA [5], it can be concluded that LA was not the underlying cause in this patient.

Given the isolated factor X deficiency, the lack of response to vitamin K and PCC treatment, and the results of mixing studies supporting the presence of an inhibitor, a specific antibody against factor X was then suspected as the underlying cause of bleeding.

To confirm this hypothesis and establish the presence of a circulating inhibitor directed against factor X, diagnostic tests were performed, using plasma dilution and mixing studies, coupled with factor X assays. These revealed a clear inhibitory effect, providing compelling evidence for the presence of the inhibitor (Table 4). The quantification of the inhibitor was determined using the Bethesda method, where a unit is defined as the amount of inhibitor that will neutralize 50% of 1 unit of factor in normal plasma after 2 hours of incubation at 37°C. The Bethesda method yielded a result of 0.4 Bethesda units per milliliter (BU/mL), suggesting a low level of inhibitor activity *in vitro*. These findings supported the likelihood of a circulating autoantibody specifically targeting factor X. Notably, a dissociation between the biological and clinical activities of the inhibitor was observed, warranting detailed exploration in subsequent sections.

### 2.3. Treatment of Factor X Inhibitor and Clinical Follow-Up

The patient's bleeding diathesis presented a significant therapeutic challenge due to the presence of this highly unusual antibody specifically targeting factor X. During the diagnostic workup, the patient received standard treatments including vitamin K, PCC, and fresh frozen plasma (FFP); however, no clinical or biological improvement was observed with these interventions. Consequently, immunosuppressive therapy was deemed necessary, and methylprednisolone was initiated at a dose of 1 mg/kg/day based on a comprehensive literature search, which will be further discussed.

The administration of corticosteroid therapy resulted in the rapid normalization of coagulation tests, effectively resolving the patient's bleeding symptoms. Simultaneously, the patient's pneumonia showed favorable progression with the prescribed antibiotic treatment.

During the follow-up examinations at 3 months, 6 months, and 1 year, no signs of recurrence were observed. At the 3-month follow-up, all coagulation tests were within the normal range, definitively ruling out LA. The corticosteroid dosage was gradually tapered during this period based on clinical judgment.

## 3. Discussion

### 3.1. Causes for Factor X Deficiencies

Acquired deficiencies in factor X often involve multiple factors, such as in cases of liver diseases or vitamin K deficiencies [1]. On the other hand, isolated factor X deficiency is typically congenital, known as Stuart–Prower disease, with an incidence of approximately 1 in 1,500,000 [2]. However, AiF10D is an extremely rare condition, with only a few reported cases in the literature. Notably, among these reported cases, the majority are associated with AL amyloidosis or plasma cell dyscrasia [2, 7]. Instances of AiF10D without amyloidosis are exceptionally rare, with only a few documented cases.

### 3.2. Specificity of Factor X Inhibitor

Among the reported cases of AiF10D, only a minority have provided compelling evidence of a circulating inhibitor specifically targeting factor X, strongly suggesting an association with factor X-specific autoantibodies. Notably, a recent literature review conducted in 2021 identified a total of 28 cases with a potential inhibitor [8]. Among these cases, 17 clearly demonstrated the presence of the inhibitor through immunological anti-factor X antibody detection or by observing an inhibitory pattern in mixing tests, which is consistent with our own case. Since this review, our own literature screening has revealed four additional cases [9, 10].

It is important to note that the presence of inhibitors can be attributed to both neutralizing and non-neutralizing anti-factor X autoantibodies. However, non-neutralizing antibodies cannot be detected by conventional assays for functional factor X inhibitors [8], which may explain the discrepancy between suspected and confirmed cases of inhibitors. We could not confirm the nature of the inhibitor as an antibody in our case due to the lack of immunological confirmation, the recommended method for accurate identification of inhibitors [8, 10]. Nevertheless, the presence of an inhibitor against factor X remains significant as it strongly suggests the involvement of factor X-specific autoantibodies.

### 3.3. Underlying Diseases and Etiological Factors

Apart from the cases of factor X deficiency associated with amyloidosis, which are thought to be due to an adsorption by amyloid fibrils [2, 6], there are various other underlying diseases associated with AiF10D. Respiratory infections, as observed in our case and reported in the literature, have been frequently associated with AiF10D. In addition, other underlying diseases such as severe burns, leprosy, inflammatory bowel diseases, and toxic insults have been reported [7, 8]. Approximately one-third of cases remain idiopathic. Interestingly, two recent cases described a factor X deficit following SARS-CoV-2 infection, although they could not demonstrate an inhibitory effect [11, 12].

Literature reports suggest that acute respiratory infections can trigger the production of infectious-related autoantibodies targeting shared epitopes between the infectious agent and factor X [2, 13].

### 3.4. A Possible Autoimmune Overlap

We also need to consider the potential autoimmune overlap in this case, given the patient's concurrent autoimmune conditions of diabetes and vitiligo. In the context of autoimmune coagulopathies, an increased association with autoimmune pathologies has been observed in cases involving anti-FVIII, FV, and FXI [14–16]. However, to date, no specific syndrome has been described that links diverse autoimmune conditions such as diabetes, vitiligo, and autoantibodies against any coagulation factors. Furthermore, there have been no previously reported cases regarding the coexistence of a factor X-related coagulopathy with other autoimmune conditions.

### 3.5. Transient Nature of Autoantibodies and Clinical Implications

In all reported cases of acquired factor X circulating autoantibodies, these antibodies have shown a transient nature without subsequent recurrence, suggesting a self-limited nature of the disease. In addition, studies have found no significant correlation between the decreased factor X activity, bleeding symptoms, and the factor X inhibitor titer [7, 8]. It is worth mentioning that the factor X inhibitor titer was determined in a limited number of cases using the Bethesda method. In a majority of these cases, the values were below 1 BU/mL [8], which aligns with our own case where the titer was measured at 0.4 BU/mL.

### 3.6. Therapeutic Approaches and Disease Evolution

In cases where AiF10D inhibitors were identified, various hemostatic therapies were administered. However, the effectiveness of treatments such as FFP and vitamin K was limited. PCC showed slightly better results, likely attributed to their higher factor X concentration, but still exhibited suboptimal outcomes in terms of coagulation parameters and bleeding control [7, 8]. The use of antifibrinolytic agents is infrequent and not strongly recommended, as AiF10D patients typically do not present a hyperfibrinolytic state. Although promising treatments, such as a high-purity plasma-derived factor X concentrate or a product combining activated factors VII and X, have been proposed [8], there is currently a lack of reported experience with their application in this specific context.

Immunosuppressive treatments, including corticoids, rituximab, cyclophosphamide, plasma exchanges, and intravenous immunoglobulin, have been used in many cases [8]. In our case, we administered methylprednisolone at a dosage of 1 mg/kg/day, following the common approach of using glucocorticoids as a primary treatment, and observed a prompt recovery. This is consistent with positive outcomes observed in similar cases [13, 17, 18]. It is also worth mentioning that in a few reported cases, spontaneous normalization of coagulation testing has been observed without the use of immunosuppressive measures [7].

Considering the general evolution of the disease, most patients achieve complete resolution, and partial resolution was observed in one case [8]. Two deaths have been reported [6, 19]. No instances of relapse have been documented in the literature thus far, suggesting the favorable prognosis of this condition.

## 4. Conclusion

In conclusion, we presented a rare case of bleeding diathesis in a patient with community-acquired pneumonia, which was found to be caused by an acquired circulating inhibitor targeting factor X. Based on the literature and clinical findings, this inhibitor is highly suspected to be an autoantibody. Prompt treatment with methylprednisolone effectively controlled the inhibitor, leading to the resolution of bleeding symptoms. This case highlights the diagnostic strategies, differential algorithm, and therapeutic approaches for managing this rare coagulopathy. Further research and experience are needed to optimize the treatment options for acquired factor X deficiency with circulating inhibitors.

## Figures and Tables

**Table 1:** Coagulation and hematological values on hospital admission.

Parameters^*∗*^	Laboratory results	Reference intervals
Hemoglobin (g/dl)	15.2	13–17
White blood cell (×10^9^/L)	6.0	4.0–10.0
Platelet count (×10^9^/L)	180	150–400
PT value (%)^†^	18	70–110
INR	4.55	0.8–1.20
PT (s)^†^	39.9	MNPT of 12.5^‡^
aPTT (s)	83.4	28–42
Thrombin time (s)	16.4	14–25
Fibrinogen (mg/dL)	386	150–400
D-dimer (mg/L FEU)^§^	0.28	0–0.50
Coagulation factor assays^||^		
Factor II (%)	62	50–150
Factor V (%)	91	50–150
Factor VII (%)	71	50–150
Factor VIII (%)	111	50–150
Factor IX (%)	121	50–150
Factor X (%)	18	50–150
Factor XI (%)	123	50–150
Factor XII (%)	82	50–150

^*∗*^Analyzers used in this hospital's laboratory are as follows: XE-5000 (Sysmex) for complete blood count, STA-R Evolution (Stago) for routine coagulation testing, and ACL TOP 500 (Werfen/Instrumentation Laboratory) for coagulation factors. ^†^Note that when using the STA-R Evolution (Stago) analyzer, a PT value expressed in % is obtained, representing the patient's coagulation time relative to the standardized coagulation times of control samples. To provide a more representative measurement, PT value in seconds (39.9) has been reported and can be compared to the MNPT (mean normal prothrombin time) value of the lab (12.5), for this lot of thromboplastin reagent. The reagent lot used for the PT determination was STA Neoplastine CI Plus 5 250576 (International Sensitivity Index (ISI) of 1.30). ^‡^MNPT represents the average prothrombin time of healthy individuals and serves as a reference for comparison for calculation of INR. ^§^Reagent used for D-dimer was STA Liatest DDi (Stago). Regarding the reference interval, 0.50 represents the cutoff value, with values greater than 0.50 mg/L FEU not supporting the exclusion of venous thromboembolism. ^||^For the determination of factor activities, the following reagents were used. PT reagent: HemosIL RecombiPlasTin (factors II, V, VII, and X). aPTT reagent: HemosIL SynthASil (factors VIII, IX, XI, and XII). These were utilized on an ACL TOP 500 automated coagulation analyzer (Werfen/Instrumentation Laboratory).

**Table 2:** Causes for abnormal prothrombin time and prolonged activated partial thromboplastin time.

Common medical states
Liver diseases
Disseminated intravascular coagulation
Vitamin K deficiency
Extensive blood product transfusion
Anticoagulant therapy

*Factor deficiencies*
Deficiency in single or multiple factors X, V, II
Antibodies against these factors
Pan-inhibitors (e.g., lupus anticoagulant, immunoglobulin)
Dysfibrinogenemia, hypofibrinogenemia

*Presence of an inhibitor*
Circulating immunoglobulin
Lupus anticoagulant
Heparin contamination

*Preanalytical factors*
Prolonged sample storage
Clotted sample
Heparin contamination
Relative excess of citrate/plasma (polycythemia, insufficient tube filling)

**Table 3:** Plasma mixing study.

	Normal pool	1/2 dilution	1/4 dilution	1/8 dilution	Patient	Reference intervals
PT (%)^*∗*^	100%	65%	88%	94%	18%	70–110
aPTT (s)^†^	33.3	45.9	36.0	33.8	81.0	28–42
aPTT (s)^‡^	30.0	33.5	31.0	30.2	53.4	28–37

^*∗*^As in Table 1, reagent used for PT determination was STA Neoplastine CI Plus 5. ^†^Mixing study was performed using two different reagents for determining aPTT. First line was using reagent STA-PTT Automate (Stago), activator being micronized silica. The aPTT value of 81.0 s in this first line, obtained from a repeated sample, exhibits a slight variance from the corresponding value in Table 1. ^‡^Second line displays aPTT determination using reagent STA-CK Prest 5 (Stago), activator being kaolin.

**Table 4:** Plasma mixing study, coupled with factor X assays.

	Normal pool (%)	1/4 dilution (%)	1/2 dilution (%)	3/4 dilution (%)	Patient (%)
Room temperature	98.50	73.30	44.90	23.10	5.40
Expected results		75.20	51.90	28.70	
Incubation for 2 hours at 37°C	109.00	69.00	47.70	21.50	4.40
Expected results		82.80	56.70	30.60	

## Data Availability

The data used to support the findings of this case are available from the corresponding author upon request.
